# Systematic Identification of Genes that Regulate Neuronal Wiring in the *Drosophila* Visual System

**DOI:** 10.1371/journal.pgen.1000085

**Published:** 2008-05-30

**Authors:** Jürg Berger, Kirsten-André Senti, Gabriele Senti, Timothy P. Newsome, Bengt Åsling, Barry J. Dickson, Takashi Suzuki

**Affiliations:** 1 Max Planck Institute of Neurobiology, Martinsried, Germany; 2 Research Institute of Molecular Pathology (IMP), Vienna, Austria; 3 Department of Developmental Biology, Wenner Gren Institute, Stockholm University, Stockholm, Sweden; 4 Karolinska Institute, Department of Biosciences and Nutrition, Section of Natural Sciences, Södertörn University College, Huddinge, Sweden; 5 School of Molecular and Microbial Biosciences, University of Sydney, Australia; 6 AstraZeneca R&D, Mölndal, Sweden; Howard Hughes Medical Institute, Northwestern University, United States of America

## Abstract

Forward genetic screens in model organisms are an attractive means to identify those genes involved in any complex biological process, including neural circuit assembly. Although mutagenesis screens are readily performed to saturation, gene identification rarely is, being limited by the considerable effort generally required for positional cloning. Here, we apply a systematic positional cloning strategy to identify many of the genes required for neuronal wiring in the *Drosophila* visual system. From a large-scale forward genetic screen selecting for visual system wiring defects with a normal retinal pattern, we recovered 122 mutations in 42 genetic loci. For 6 of these loci, the underlying genetic lesions were previously identified using traditional methods. Using SNP-based mapping approaches, we have now identified 30 additional genes. Neuronal phenotypes have not previously been reported for 20 of these genes, and no mutant phenotype has been previously described for 5 genes. The genes encode a variety of proteins implicated in cellular processes such as gene regulation, cytoskeletal dynamics, axonal transport, and cell signalling. We conducted a comprehensive phenotypic analysis of 35 genes, scoring wiring defects according to 33 criteria. This work demonstrates the feasibility of combining large-scale gene identification with large-scale mutagenesis in Drosophila, and provides a comprehensive overview of the molecular mechanisms that regulate visual system wiring.

## Author Summary

In the nervous system, every neuronal process should know where to grow and when to establish contacts to the next-order neurons. During development, it is known that neural circuit formation is primarily determined by the genes. To identify these genes, we focused on the *Drosophila* visual circuitry as a model system, and disrupted the genes randomly. From over 30,000 of these mutants, we found more than 100 mutants which have disrupted patterns of neural circuitry, which we assessed as representing about 40 genes. We have successfully nailed down which gene is disrupted in 36 of them. We provide a list of all of the genes we identified. Altogether, we performed a detailed characterization of the 35 mutant phenotypes, to assess which aspects of neural circuit formation are disrupted in each of the mutants. Summarizing and categorizing the phenotypic fingerprints of each mutant, we could see which genes are more closely related to the others. These data will be useful for clarifying the genetic program that controls neural circuit formation, not only for the *Drosophila* visual system, but also generally for nervous systems across the species.

## Introduction

The adult visual system of *Drosophila melanogaster* is a powerful genetic model for exploring the molecular and cellular mechanisms involved in axon growth, guidance, and synaptic specificity [1]. The adult retina consists of some 800 ommatidia, each containing 8 photoreceptor cells (R1–R8) that form topographic connections in distinct layers of the optic lobe. These connections are established during the late larval and early pupal stages. As photoreceptors begin to differentiate in the eye imaginal disc, the R1–R8 axons from each ommatidium form a single fascicle that extends topographically into the brain. Within the optic lobe, the R1–R8 axons then defasciculate and select their individual target regions. R1–R6 cells connect to targets in the lamina region of the optic lobe as part of a circuit specialized for motion detection. R7 and R8 cells, which mediate color vision, project axons through the lamina to terminate in distinct layers of the underlying medulla.

Large-scale forward genetic screens have been used to isolate numerous mutations disrupting various aspects of visual system wiring [2]–[5]. A small subset of these mutations has been selected for positional cloning, and the genes thus identified have provided important entry points for further mechanistic studies [6]. As with most large-scale genetic screens performed in *Drosophila*, the selection of mutations for gene identification has often been made on an ad hoc basis. In many cases, selection has been guided in part by the strength and specificity of the mutant phenotype, but also rather opportunistically by the number of alleles recovered and any prior genetic information that might facilitate the challenging task of positional cloning.

For these reasons, the potential of this model system has not yet been fully exploited. In particular, the bias for strong and specific mutant phenotypes has evidently enriched for genes encoding regulatory proteins such as transcription factors and cell surface receptors. Mutations affecting the basic machinery of axon growth, guidance, and targeting are likely to result in more pleiotropic defects. Additionally, because of protein perdurance and possible genetic redundancy, mutations in such genes may not always lead to a pronounced wiring defect. For these reasons, we were motivated to take a more systematic approach to gene identification–one that would be robust enough to identify even those genes with only one mutant allele, and efficient enough to justify identifying those with less specific or less potent mutant phenotypes. Accordingly, we developed methods for genetic mapping using single nucleotide polymorphisms (SNPs) [7]. We have now used these methods to systematically identify the gene disrupted for nearly all the mutations recovered in a large-scale forward genetic screen for visual system connectivity defects.

## Results/Discussion

### Isolation of Mutations that Disrupt Visual System Wiring

Using *eyFLP* to generate whole-eye clones [4], we screened each of the four major autosomal arms for chemically-induced mutations that disrupt visual system wiring. Eye-Brain complexes were dissected from 3^rd^ instar larvae harbouring the *glass-lacZ* reporter [8], fixed and stained by X-gal to visualize R-axon projections. Specimens were examined under a light stereomicroscope. Lines exhibiting aberrantly patterned retinas, as assessed initially from the external morphology of the adult eye and subsequently from tangential sections, were not further processed. Thus, we retained only those mutants in which the R cells appear to be appropriately specified, but their axons do not project correctly within the optic lobe [4]. Ultimately, we retained 122 mutants from a total of 32,175 lines screened (Table 1). Sporadic transheteroallelic larval or adult survivors were tested for phenotypic non-complementation either by staining of 3^rd^ instar larval eye-brain complexes or horizontal adult head sections, respectively. Additionally, we analysed the R-cell projections in adult *ey*FLP mosaics of each complementation group by staining horizontal head sections to test the phenotypic consistency within the group. On this basis, mutant lines were assigned to 42 loci, 21 of which are represented by multiple alleles (Table 1).

**Table 1:** Identification of genes required for visual system wiring.

Chromosome arm	Lines screened	Mutations recovered	Number of loci	Genes identified
2L	7,319	23	10	9
2R	9,781	32	9	9
3L	7,006	32	15	11
3R	8,069	35	8	7
**Total**	**32,175**	**122**	**42**	**36**

### Systematic Positional Cloning

Six genes were identified using standard positional cloning procedures, and have been reported previously [4], [9]–[12]. For the remaining loci, we used SNP mapping to identify the relevant gene [7]. The strategy was to isolate a set of ∼50 recombinants between the mutant and a reference chromosome, selecting for recombination events across the entire chromosome arm. Each of these recombinants was then scored for a visual system wiring phenotype (in *eyFLP* clones) and for SNP genotypes. This typically mapped the mutation to an interval of 0.5–1.5 Mb. In a second phase, a further set of 100–200 recombinants was generated within this interval, usually using a pair of flanking P-element insertions as markers. This second set of recombinants was also scored for a visual system wiring defect and SNP genotype. In some cases, rather than mapping the visual system phenotype at this second stage, we alternatively tracked a lethal mutation within this narrower interval (assuming the two to be due to the same genetic lesion). In these cases, we generated around 100 recombinants each from two P element insertion lines that were flanking the interval. This procedure gave a resolution of approximately 10–30 kb. Finally, we sequenced predicted coding regions in this region, using genomic DNA extracted from homozygous mutant and control embryos (see materials and methods). In some cases, the mutant gene was identified by a failure to complement existing alleles, in tests performed at various stages during the mapping procedure. Whenever possible, complementation was confirmed by examining visual system wiring in trans-heteroallelic animals.

Using these procedures we were able to identify a further 30 genes, two of which we have previously reported [7] and 28 of which are described here. For 12 of these loci, the gene identification was confirmed in a rescue experiment, generating transgenic animals carrying either a cDNA under the control of the eye-specific *GMR* or *eyeless* promoter, or inserting a genomic fragment. In total, we have now identified 36 of the 42 genes identified in this screen, including six genes identified by standard positional cloning. These genes are listed in Table 2, along with a summary of the evidence supporting each assignment. Of these 36 genes, visual system wiring defects have previously been reported for 11 loci: *brakeless, dead-ringer*/*retained*, *dock*, *flamingo*, *misshapen*, *LAR*, *N-Cadherin*, *Pak*, *Ptp69D*, *golden goal* and *trio* [3], [4], [7], [9]–[20]. Another 5 genes have been reported to have neuronal phenotypes in other developmental processes: *chickadee*, *enoki mushroom*, *kinesin heavy chain*, *unc-104* and *sequoia* [21]–[26]. The remaining 20 genes have not previously been associated with neural phenotypes and for five of these no mutations have previously been reported (*Br140*, *cdk8*, *wnk*, *ckIIα*, *GUK*-*holder*).

**Table 2:** Genes required for visual system wiring.

Gene	CG number	Arm	Location^1^	*Alleles*	*Evidence*				Rescue^4^	Mutations^5^	Reference^6^
					Fails to complement existing alleles		SNP mapping				
					Lethality^2^	Visual system^3^	Lethality^2^	Visual system^3^			
*kismet*	CG3696	2L	211k..251k	7	Yes					N.D.	
*dock*	CG3727	2L	826k..833k	1	Yes	Yes				See ref.	9
*chickadee*	CG9553	2L	5,973k..5,981k	1	Yes	Yes		5,958k..6,238k		G1338:L71F	
*Hrb27c*	CG10377	2L	6,921k..6,926k	1	Yes	Yes		6,914k..6,969k		E752:C82W	
*taiman*	CG1310	2L	9,167k..9,246k	1	Yes	Yes	∼9,245k	8,040k..9,603k		N.D.	
*basket/JNK*	CG5680	2L	10,247k..10,250k	1	Yes			10,221k..10,267k		H15:I212F (PB)	
*N-Cadherin*	CG7100	2L	17,646k..17,735k	5	Yes	Yes				N.D.	
*brat*	CG10719	2L	19,130k..19,169k	1	Yes		∼19,164k	18,458k..19,560k	Yes	K1771:Δ499bp^7^	
*LAR*	CG10443	2L	19,607k..19,727k	4	Yes	Yes			See ref.	See ref.	12
*Br140*	CG1845	2R	2,784k..2,790k	2	Yes				Yes	S781:W325stop S203:P1320L	
*flamingo*	CG11895	2R	6,222k..6,237k	9	Yes	Yes			See ref.	N.D.	10
*Psc*	CG3886	2R	8,482k..8,497k	1	Yes			8,291k..8,861k		N.D.	
*sequoia*	CG17724	2R	8,696k..8,706k	2	Yes			8,528k..9,195k		A436:Q713stop G25:Q620stop	
*khc*	CG7765	2R	11,782k..11,787k	2	Yes^8^					N.D.	
*unc-104*	CG8566	2R	12,266k..12,281k	10			∼12,180k..12,310k^9^		Yes	R1829:34splice^10 ^R403:N316I P350:W606stop C674:W709stop R767:W727stop R757:Q990stop^11^	
*brakeless*	CG5580	2R	13,794k..13,805k	2	Yes				See ref.	See ref.	11
*dead-ringer*	CG5403	2R	19,140k..19,162k	2	Yes	Yes	∼19,161k		Yes	J1609:Δ276bp^7^ R971:Δ1005bp^7^	7
*enok*	CG11290	2R	19,607k..19,615k	2	Yes	Yes			Yes	K1293:R365stop Q253:L872stop	
*trio*	CG18214	3L	980k..1,018k	10	Yes	Yes			See ref.	See ref.	9
*misshapen*	CG16973	3L	2,539k..2,570k	1	Yes			2,502k..2,671k		N.D.	7
*archipelago*	CG15010	3L	4,230k..4,238k	1			4,205k..4,261k	3,546k..4,322k		F387:Q1195stop	
*Klp64D*	CG10642	3L	5,331k..5,333k	1	Yes			5,283k..5,350k		N.D.	
*Cdk8*	CG10572	3L	9,811k..9,813k	1			9,806k..9,816k	9,569k..10,175k	Yes	H2480:P154L	
*Ptp69D*	CG10975	3L	12,709k..12,716k	3	Yes	Yes			See ref.	See ref.	4
*Mbs*	CG32156	3L	16,017k..16,047k	1	Yes	Yes		15,433k..17,428k		N.D.	
*non-stop*	CG4166	3L	18,584k..18,587k	2	Yes		∼18,587k	17,652k..19,075k	Yes	F1935:G688D	
*golden goal*	CG32227	3L	20,203k..20,229k	3			20,108k..20,218k		Yes	D869:A670splice^12^ D1600:Q573splice^12^ H1675:Q255stop	20
*wnk*	CG7177	3L	21,478k..21,489k	3			∼21,477k		Yes^13^	G1286:S529F F1183:Q1151stop D482:Q2026stop	
*ckIIα*	CG17520	3L	23,033k..23,037k	2				>22,887k	Yes	H3091:D212N G703:W279G	
*Xe7*	CG2179	3R	1,491k..1,495k	1	Yes	Yes		1,452k..1,548k		J1828:Y495stop	
*Pak*	CG10295	3R	2,177k..2,186k	12	Yes	Yes				See ref.	9
*Sim*	CG7771	3R	8,883k..8,903k	1	Yes^14^			8,717k..9,182k		N.D.	
*trithorax*	CG8651	3R	10,089k..10,113k	7	Yes^8^					N.D.	
*GUK-holder*	CG31043	3R	14,810k..14,848k	5				14,794k..14,941k	Yes	J785:Q520stop J1024:W596stop I256:W735stop	
*bonus*	CG5206	3R	16,419k..16,439k	1	Yes			15,145k..17,211k		N.D.	
*headcase*	CG15532	3R	26,104k..26,188k	7	Yes	Yes	∼26,128k		Yes	N.D.	

1 Locations according to *Drosophila* genome sequence Release 4.2.1.

2 Mapping based on lethality of trans-heterozygous allele combinations.

3 Mapping based on visual system wiring phenotype in trans-heterozygous combinations or in whole-eye clones.

4 Rescue of visual system wiring phenotype with a *GMR* transgene.

5 Identified mutations, showing allele name followed by predicted amino acid or other mutation (based on PA isoform, unless otherwise indicated).

6 Previous detailed report of alleles recovered in this screen.

7 the number indicates the size of the deletions in nucleotides.

8 Mapped using chromosomal deficiencies.

9 Mapped using P-element induced male recombination.

10 Splicing consensus site was mutated which resulted in complete failure of proper splicing.

11 Based on Genbank protein sequence AAF74192.

12 splicing donor site was mutated leading to a premature stop before the transmembrane domain Please see ref [20] for the further detail.

13 Rescue using genomic fragment.

14 Fails to complement *sim* embryonic CNS phenotype.

### Phenotypic Classification of Wiring Mutants

In parallel with the systematic gene identification, we also performed a comprehensive phenotypic analysis of all mutant loci, selecting one or two representative alleles for those loci with multiple alleles. Our objective was to obtain an unbiased and semi-quantitative description of visual system wiring defects in each mutant as guide for future phenotypic and molecular studies. The screen was performed with a general R axon marker (*glass*-*lacZ*), which provides only low information content, we therefore examined each mutant using a panel of additional R-cell class-specific markers—*Rh1*-*τlacZ* (R1–R6 axons), *Rh4*-*mCD8:GFP* (R7 axons), *Rh6*-*mCD8:GFP* (R8 axons), and *omb*-*τlacZ* (polar axons)—as well as the additional general R-axon marker anti-Chaoptin mAb24B10. For each marker and mutant, visual system wiring was examined in whole-eye *eyFLP* clones in either 3rd instar larvae (*glass-lacZ* and *omb-τlacZ*) or in adults (*Rh1*-*τlacZ*, *Rh4*-*mCD8:GFP*, *Rh6*-*mCD8:GFP*, and mAb24B10). A total of 33 criteria of wiring defect were identified (Table S1, Table S2), and each defect was scored for each mutant using a scale of 0 (no defect) to 4 (most severe).

The *dock* allele D333 was excluded from our phenotypic analysis as molecular data [9], previously published reports [14] and complementation analysis suggests that it is a weak hypomorph.

For each of the data point (A score for each defect criterion of each mutant line), 2–5 hemispheres from multiple eye-brain complexes were scored independently by two investigators (T.S. and J.B.), generally from confocal microscope images. The two investigators score the same images. Wherever the larger sample size examination was possible, we prepared more than 10 samples to assess more reliably the expressivity and the penetrance of the phenotypes (e.g. *omb-τlacZ* (polar axons), adult *gl-lacZ* section and *Rh1-τlacZ* sections). For the confocal samples which we appreciated the resolution quality of the images that were taken, we assessed the expressivity by calculating the difference between the highest and the lowest score given within each defect criterion for each mutant allele. This reflects the variation of the scores we obtained and will help understand the expressivity of the each phenotype in each mutant allele (Figure S1). We also demonstrate the penetrance of the phenotype by checking whether each defect criterion was “fully penetrant” in our analysis (Figure S1).

**Figure 1:**
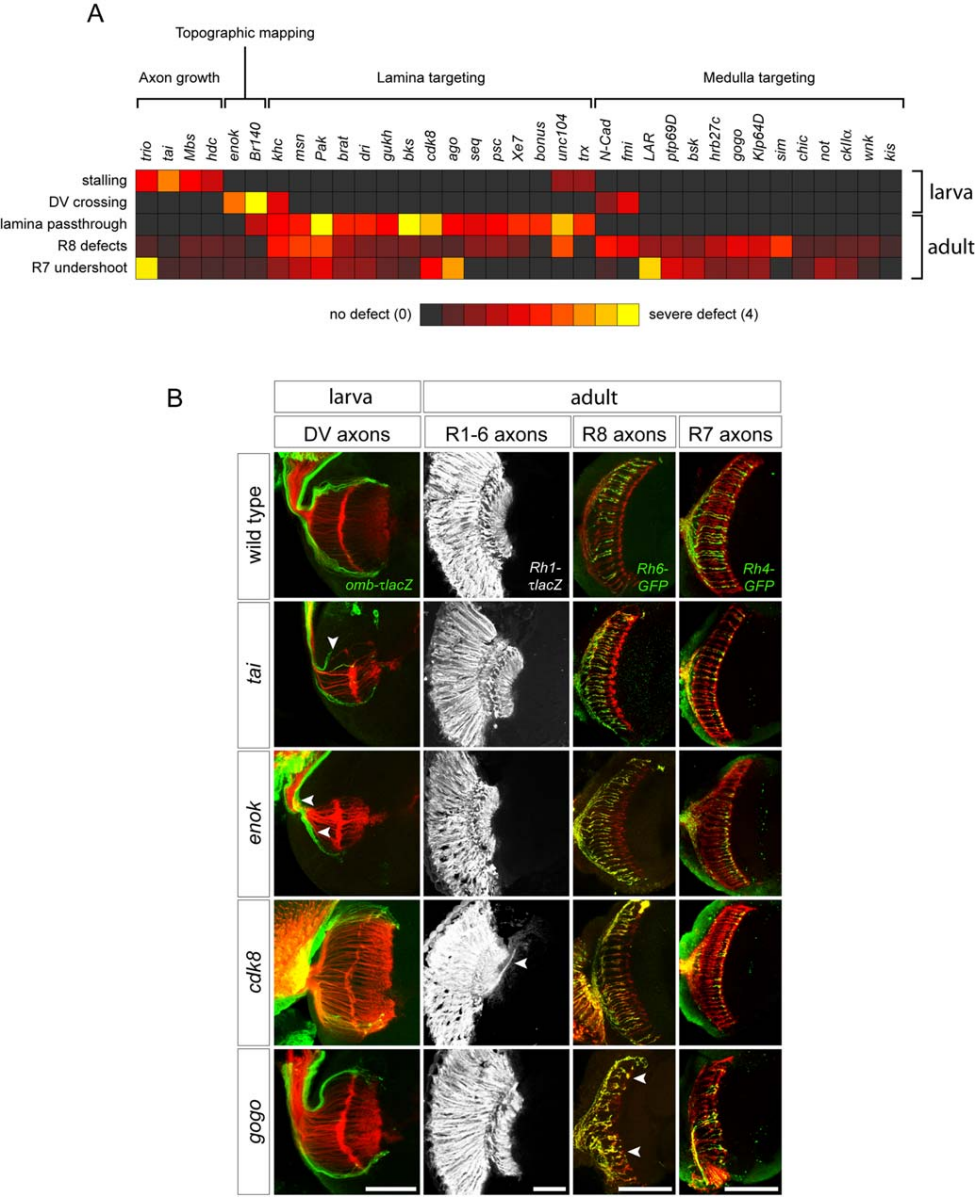
Classification of visual system wiring mutants. (A) Diagnostic phenotypic defects for the four major mutant classes, scored on a scale from 0 (no defect, black) to 4 (most severe defect, yellow). “R8 defects” is an average of all R8 phenotypes (Table S1). (B) Wild-type visual system anatomy and examples of mutants in each class. From left to right, images show: DV axons, whole-mount larval eye-brain complexes stained with mAb24B10 to visualize all R-axons (red) and anti-β-galactosidase to visualize dorsal and ventral axons expressing an *omb-τlacZ* reporter (green); R1–R6 axons, adult brain sections stained with anti-β-galactosidase to visualize R1–R6 axons expressing an *Rh1-τlacZ* reporter; R8-axons, confocal sections of adult brains stained with mAb24B10 (red) and anti-GFP to visualize R8-axons expressing an *Rh6-GFP* reporter (green); R7-axons, confocal sections of adult brains stained with mAb24B10 (red) and anti-GFP to visualize R7 axons expressing an *Rh4-GFP* reporter (green). *tai* and *enok* illustrate stalling and ventral mistargeting of dorsal *omb-τlacZ* axons, respectively (arrowheads). In *cdk8* clones, some R1–R6 axons project through the lamina and across the optic chiasm into the medulla (arrowhead). R8-and R7-axons are disorganized in *gogo* clones, and some R8-axons extend to the R7 target layer (arrowheads). For the larval eye-brain complexes, dorsal is up and anterior left; for adult brain sections, anterior is up and lateral left. Scale bars, 50 µm.

For classifying the mutants, we took advantage of hierarchical clustering method. Instead of a single clustering based on all 33 defect criteria, we first selected five prominent defect criteria that gave an informative primary classification of the mutants (Figure 1). These 5 defects are axon stalling, dorsal-ventral (DV) crossing, lamina pass-through, R8 defects, and R7 undershoot. Although many mutants have more than one of these defects, these phenotypes could nevertheless be used to classify the mutants into 4 major phenotypic clusters, each representing a distinct biological step in visual system wiring: axon growth, topographic mapping, lamina targeting, and medulla targeting. With this procedure, we put more weight on these selected five criteria, which we consider of high biological importance. In the following sections, we provide a brief overview of the genes and phenotypes in each of these 4 classes, considering the full set of 33 defect criteria.

### Axon Growth

**Figure 2:**
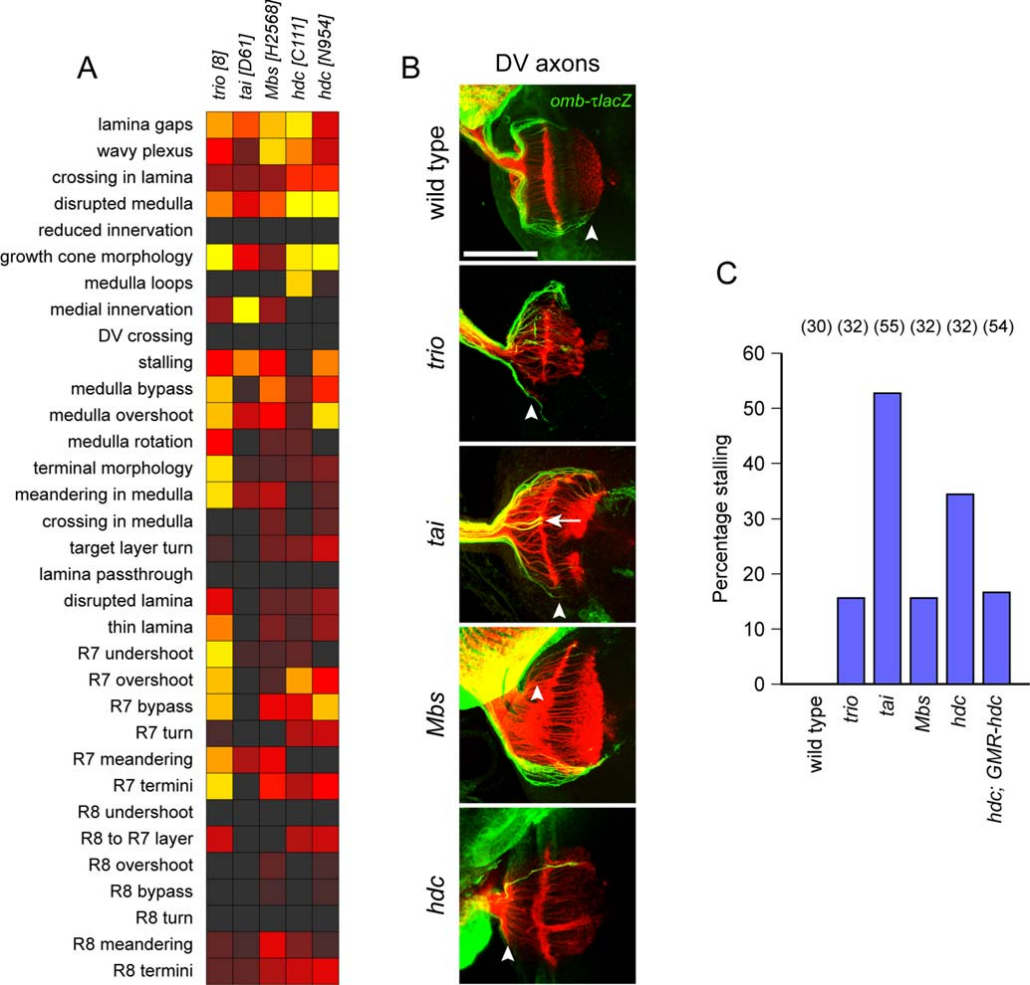
Axon growth genes. (A) Full phenotypic analysis of mutants in the axon growth class, scored for all defect criteria as in Figure 1A. (B) Whole-mount larval eye-brain complexes stained with mAb24B10 to visualize all R-axons (red) and anti-β-galactosidase to visualize dorsal and ventral axons expressing an *omb-τlacZ* reporter (green). Arrowheads indicate delayed or stalled axons; arrow indicates polar axons misrouted to the equatorial regions of the optic lobe. Scale bar, 50 µm. (C) Quantification of stalling defects, scored by counting the percentage of larval eye-brain complexes in which at least some *omb-τlacZ* axons failed to extend fully within the optic lobe, as visualized by X-gal stainings, (*n*).

Mutations in four genes resulted in a characteristic stalling phenotype, readily visualized with the *omb-τlacZ* transgene at the larval stage (Figure 2). This marker labels axons from the dorsal and ventral regions of the eye disc, which target the corresponding dorsal and ventral regions of the optic lobe. In axon growth mutants, a portion of axons appear to stall within the optic stalk, or enter the optic lobe but fail to reach their normal target region. Nevertheless, these axons generally appear to remain on course, suggesting that the defect is primarily in axon growth rather than guidance.

Two of the genes in the phenotypic cluster encode conserved regulators of cytoskeletal dynamics (*trio* and *Mbs*), another encodes a conserved cytoplasmic protein of unknown molecular function (*hdc*), and a fourth encodes a hormone receptor co-activator (*tai*). For each of these mutants, we performed a rigorous quantification of the stalling phenotype (Figure 2C). For *hdc*, a partial rescue was obtained with an eye-specific *GMR-hdc* transgene (Figure 2C); a similar rescue experiment for *trio* has been reported previously [9].

We and others have previously characterised the axon stalling defects in *trio* mutants, both in the visual system [9] and in the embryonic CNS and PNS [27]–[30]. Trio is a RhoGEF that activates the three *Drosophila* Rac GTPases, Rac1, Rac2, and Mtl. Similar axon stalling defects occur in animals that lack multiple copies of these *Rac* genes [27].

*Mbs* also encodes a cytoskeletal regulator—the regulatory myosin-binding subunit of myosin phosphatise [31],[32]. Myosin phosphatase negatively regulates myosin II through dephosphorylation of myosin regulatory light chain (MRLC). Loss of *Mbs* is predicted to result in increased actomyosin contractility and hence reduced motility. Consistent with this, *Mbs* mutations block epithelial sheet movement during embryonic dorsal closure, accompanied by an accumulation of F-actin at the leading edge [31],[32]. *Mbs* mutations have also been independently isolated in an *eyFLP* screen for R cell differentiation, and shown to result in the occasional translocation of the R cell body toward the axon terminus [33]. We did not see this defect in our allele, perhaps because it is hypomorphic. Stalling at the axon tip, like forward translocation of the cell body, may be due to increased traction within the R cell.

*hdc* encodes a cytoplasmic protein without any predicted functional domains, but with highly conserved vertebrate homologs [34]–[36]. In flies, *hdc* regulates branching of developing tracheal tubes, and is required in cells that will branch in order to inhibit branching of their neighbours [34]. Some indicative links have been made between human *hdc* homologs and cancer development [35],[37].

The fourth gene in this class, *tai*, encodes a steroid receptor co-activator related to the mammalian AIB-1 (or SRC-3), a gene that is amplified in breast cancer [38],[39]. *tai* regulates the migration of border cells in the *Drosophila* ovary, probably in response to the steroid hormone ecdysone [38]. Similarly, AIB-1 is evidently required for mammary duct outgrowth in a mouse tumor model [40]. In the *Drosophila* visual system, *tai* might similarly function in the migration of R axon growth cones, perhaps in response to the pulse of ecdysone that accompanies pupariation. Unlike the other three mutants in this class, *tai* also shows an axon guidance phenotype, in that the polar R axons labelled with *omb-τlacZ* often innervate medial regions of the optic lobe (Figures 2A, B). However, axon stalling is more frequent in *tai* than in any of the other outgrowth mutants (Figure 2C), possibly indicating that this misrouting is a secondary consequence of severe stalling defects.

### Topographic Mapping

**Figure 3 pgen-1000085-g003:**
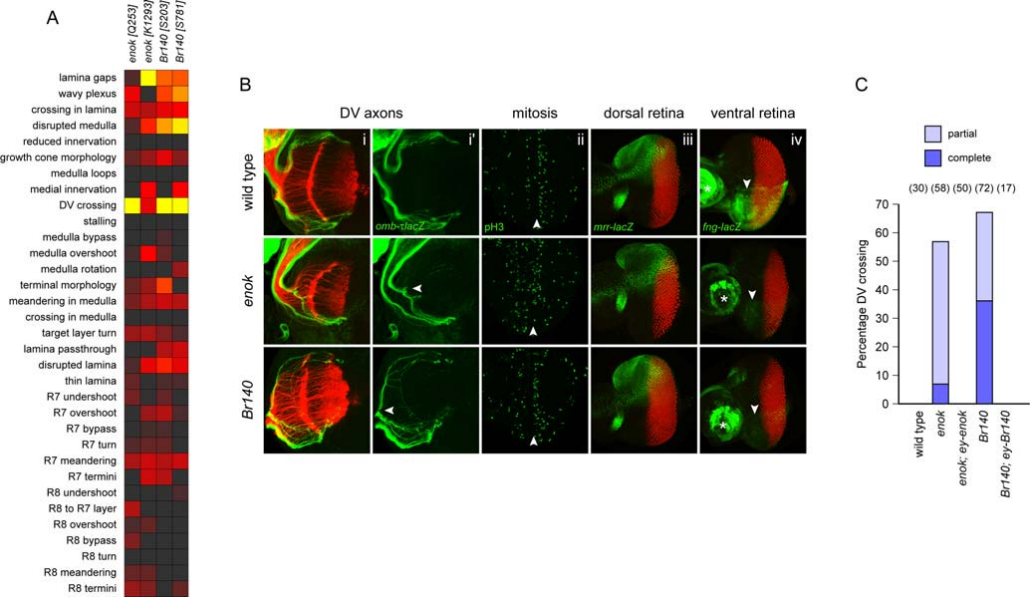
Topographic mapping genes. (A) Phenotypic analysis of *enok* and *Br140* mutations, scored for all defect criteria as in Figure 1A. (B) Whole-mount larval eye-brain complex of wild-type and *eyFLP* clones of *enok* and *Br140*. (i and i′) Staining of the optic lobe with mAb24B10 to visualize all R-axons (red) and anti-β-galactosidase to visualize dorsal and ventral axons expressing an *omb-τlacZ* reporter (green). Left panels (i) show both channels; right panels (i′) show the green channel only. Arrowheads indicate ventral misrouting of dorsal *omb-τlacZ* axons in the *enok* and *Br140* mutants, which occurs at the surface of the optic lobe. (ii) Staining of the eye disc with the mitotic marker anti-phospho H3 (green). Arrowheads indicate the position of the morphogenetic furrow. In both wild-type and mutant discs, mitotic cells are observed in a dispersed pattern ahead (left) of the furrow and in a narrow zone just behind it. (iii) Staining of the eye disc with anti-elav to visual R-cell nuclei (red) and anti-β-galactosidase to visualize dorsal cells expressing an *mrr-lacZ* reporter (green). (iv) Staining of the eye disc with anti-elav to visual R-cell nuclei (red) and anti-β-galactosidase to visualize ventral cells expressing an *fng-lacZ* reporter (green). Expression of the *fng-lacZ* reporter is greatly reduced in the *enok* and *Br140* eye discs (arrowheads), but remains in the antennal disc (asterisks). (C) Quantification of dorsal-to-ventral mistargeting, scored by counting the percentage of larval eye-brain complexes in which at least some (“partial”) or all (“complete”) dorsal *omb-τlacZ* axons projected ventrally within the optic lobe, as visualized by X-gal stainings, (*n*).

R-cell axons preserve their topographic arrangement as they project along the optic stalk and then fan out within the optic lobe. Topographic mapping along the dorsoventral axis is thought to involve both local R-cell axon–axon interactions and long-range positional cues, possibly involving molecular gradients [41],[42]. The *omb-τlacZ* marker that we used to detect axon stalling defects is an ideal marker to assess topographic mapping, as it labels the dorsal- and ventral-most R-cells in the retina and their respective projections to the dorsal and ventral regions of the optic lobe. With this marker we identified mutations in two genes with strong defects in topographic mapping: *enoki mushroom* (*enok*) and *Br140* (Figure 3A).

In mutant *eyFLP* clones for either *enok* or *Br140*, the dorsal *omb-τlacZ* axons projected aberrantly to the ventral region of the optic lobe (Figures 3B(i) and 3C). They do not appear to stall, nor innervate medial regions of the optic lobe. We infer that these dorsal axons are not impaired in their growth, nor in their ability to distinguish polar from equatorial regions of the optic lobe. Rather, they are specifically disrupted in their ability to choose a dorsal rather than a ventral trajectory. The converse defect, of ventral axons mistargeting to dorsal regions, was not observed in either mutant.

The *enok* gene encodes a putative member of the MYST family of acetyltransferases [24]. Mutations in *enok* have previously been shown to disrupt proliferation of mushroom body neuroblasts [24]. We noted that *enok* mutant eyes are sometimes reduced in size, and suspected a similar proliferation defect might also occur in the eye. However, staining with the mitotic marker anti-phospho H3 did not reveal any defects in cell proliferation (Figure 3B(ii)), and so we conclude that the function of *enok* in topographic mapping of R cell axons is unrelated to its role in cell proliferation. Our two alleles are due to nonsense mutations before and within the catalytic domain, respectively, suggesting that acetyltransferase activity is essential for topographic mapping.

Mutations in *Br140* have not been previously reported. This gene encodes a protein with predicted C2H2 zinc-finger, PHD, bromo, and PWWP domains. Bromodomains in other proteins bind acetylated lysines [43], and the close similarity of the *enok* and *Br140* phenotypes suggest that Br140 might recognize Enok substrates. Br140 proteins are highly conserved throughout evolution, including the human Br140/peregrin protein [44] and *C. elegans* LIN-49 [45].

Because mutations in both *enok* and *Br140* specifically disrupted dorsal and not ventral axon projections, we tested whether expression in the ventral retina might be sufficient to reroute ventral axons to the dorsal optic lobe. We prepared transgenes that drive expression of *enok* or *Br140* in the entire eye disc with either the *GMR* or *eyeless* promoter. Introducing these transgenes into the corresponding mutants with *eyFLP* clones restored normal targeting of dorsal axons but did not lead to dorsal mistargeting of ventral axons (Figure 3C and data not shown). We conclude from these experiments that *enok* and *Br140* are necessary but not sufficient for dorsal targeting.

To test whether dorsoventral patterning of the eye disc is also disrupted in these mutants, we examined the expression of *mirror* (*mrr*), a dorsal eye marker [46] and *fringe* (*fng*), a ventral marker [47]. We found that a *mrr-lacZ* reporter is expressed normally in the dorsal eye disc in both *enok* and *Br140* clones (Figure 3B(iii)), but the ventral expression of a *fng-lacZ* reporter was significantly reduced (Figure 3B(iv)). Loss of *fng* in the ventral eye disc does not however account for the misrouting of dorsal axons, as these axons project normally in *fng* mutant clones (not shown and [41]).

Dorsal-to-ventral targeting defects do occur in mutant clones lacking all three genes of the *Iroquois* complex (*Iro-C*), to which *mrr* belongs [41]. However, *mrr-lacZ* is still expressed normally in *enok* and *Br140* mutant clones, and *enok* and *Br140* are ubiquitously expressed in the eye disc, including the ventral regions where *Iro-C* genes are absent. Thus, we infer that *enok* and *Br140* act independently of the *Iro-C* genes in patterning the dorsal region of the eye disc, resulting in *fng* expression in the ventral region and dorsal targeting of dorsal axons.

It is also interesting to note that the reciprocal phenotype, of ventral axons targeting the dorsal region of the optic lobe, has recently been reported for mutations in *Wnt4*, *Dfrizzled2* and *dishevelled*, implicating the Wnt signalling pathway in the establishment of a ventral projection [41]. We did not recover any mutations in these genes in our screen, presumably because these mutations also disrupt eye patterning and would have been discarded in our initial analysis.

### Lamina Targeting

**Figure 4 pgen-1000085-g004:**
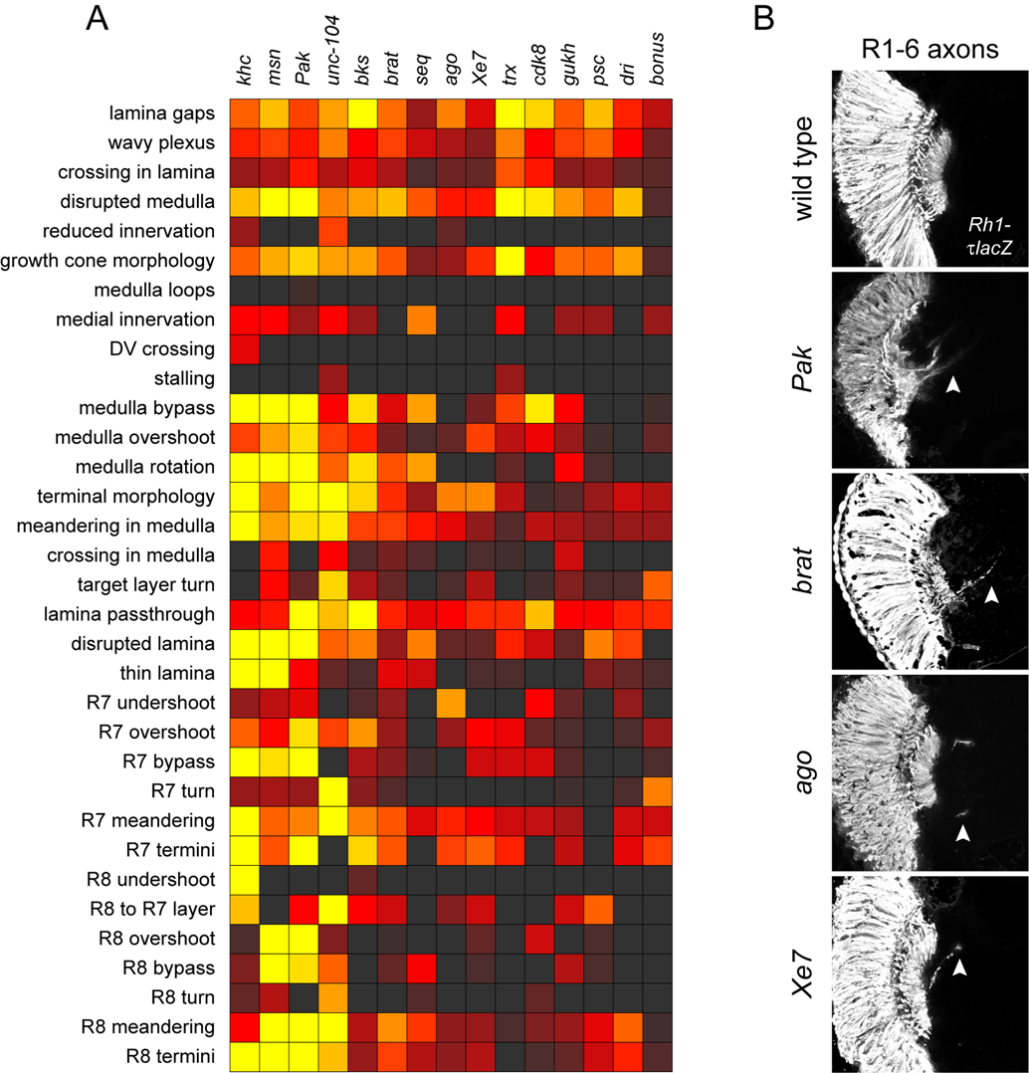
Lamina targeting genes. (A) Phenotypic analysis of mutants in the lamina targeting class, scored for all defect criteria as in Figure 1A. (B) Horizontal sections through the optic lobes of adult heads, stained with anti-β-galactosidase to visualize R1–R6 axons expressing an *Rh1-τlacZ* reporter. Arrowheads indicate R1–R6 axons extending through the lamina into the medulla in whole-eye *eyFLP* clones of selected mutants.

R1–R6 axons terminate in the lamina in response to signals from lamina glial cells, the intermediate targets for these axons. The nature of this glial signal, and how R1–R6 axons respond to it, is unknown. However, if lamina glial cells are absent or reduced in number, then R1–R6 axons continue through to the lamina [48]–[50]. Such a “lamina pass-through” phenotype is readily visualized with the marker *Rh1-τlacZ*, which labels the axonal projections of R1–R6. In our screen, we identified mutations in 15 genes that exhibit a lamina pass-through phenotype. Although they formed a well-defined phenotypic cluster in our initial analysis (Figure 1A), these mutations are generally very pleiotropic (Figure 4A), suggesting that many different types of defect may result in some R1–R6 axons missing their stop signal in the lamina.

The four genes in the lamina pass-through class with the most pleiotropic phenotypes are *kinesin heavy chain* (*khc*), *unc-104*, *Pak*, and *misshapen* (*msn*) (Figure 4A). Both *khc* and *unc-104* encode kinesins, belonging to the kinesin-1 family of conventional kinesins, and the kinesin-3 family of monomeric kinesins, respectively [26],[51],[52]. These are the major kinesin families that deliver cargo to the tips of growing axons, and so the pleiotropic wiring defects in these mutants are perhaps not surprising. Interestingly, *unc-104* has been reported to be involved in retrograde transport of neurosecretory vesicles, as well as the anterograde transport [53]. In our mutant analysis, we noticed aberrant perpendicular turn of R7 axons (Figure 4A), which is indicative of a failure in retrograde transport of Smad2 protein mediated by the *Drosophila* Activin receptor Baboon [54].

*Pak* and *msn* both encode Ste20-like serine-threonine kinases [21],[55]. The broad range of defects seen in these mutants, as reported here (Figure 4A) and previously [9],[17],[18], may reflect functions of these two kinases in diverse signaling pathways.

Another set of genes in this class encodes regulators of gene expression, including two chromatin remodelling factors (*trx, Psc*) [56],[57], four putative transcription (co-)factors (*bonus*, *brakeless* [*bks*], *dri, sequoia*) [11],[19],[25],[58],[59], an RNA polymerase II C-terminal domain kinase (*cdk8*) [60], a splicing factor (*Xe7*) [61], and a translational repressor (*brat*) [62],[63].

The two remaining genes in this phenotypic cluster do not fit neatly into a single molecular class. These are *archipelago* (*ago*) and *GUK-holder* (*gukh*). *ago* encodes an F-box protein that is the substrate-specificity unit of the SCF ubiquitin ligase, and acts as a negative regulator of cell growth [64],[65]. This raises the possibility that excessive axon growth might contribute to the R1–R6 pass-through phenotype in *ago* mutant clones. The *gukh* gene was originally isolated in a two-hybrid screen for proteins interacting with Discs Large, the *Drosophila* ortholog of the post-synaptic scaffolding protein PSD-95 [66]. *Gukh* encodes two protein isoforms, Gukh-PA and Gukh-PB, which function in synaptic bouton budding at the larval neuromuscular junction [66]. Both isoforms contain an N-terminal WASP homology domain 1 (WH1), suggesting a possible role in the regulation of actin polymerisation, as well as a C-terminal PDZ-binding motif. Proteins with a similar structure are found in other species, including the human Nance-Horan syndrome protein [67]–[69]. We isolated 3 *gukh* alleles, all associated with nonsense mutations. One is predicted to truncate both the PA and PB isoforms, whereas the other two truncate only the PA isoform. In rescue experiments using *GMR* promoter, expression of Gukh-PA in the eye disc was sufficient to fully rescue the R1–R6 lamina pass-through phenotype in *gukh* mutant clones (Table 2).

### Medulla Targeting

**Figure 5:**
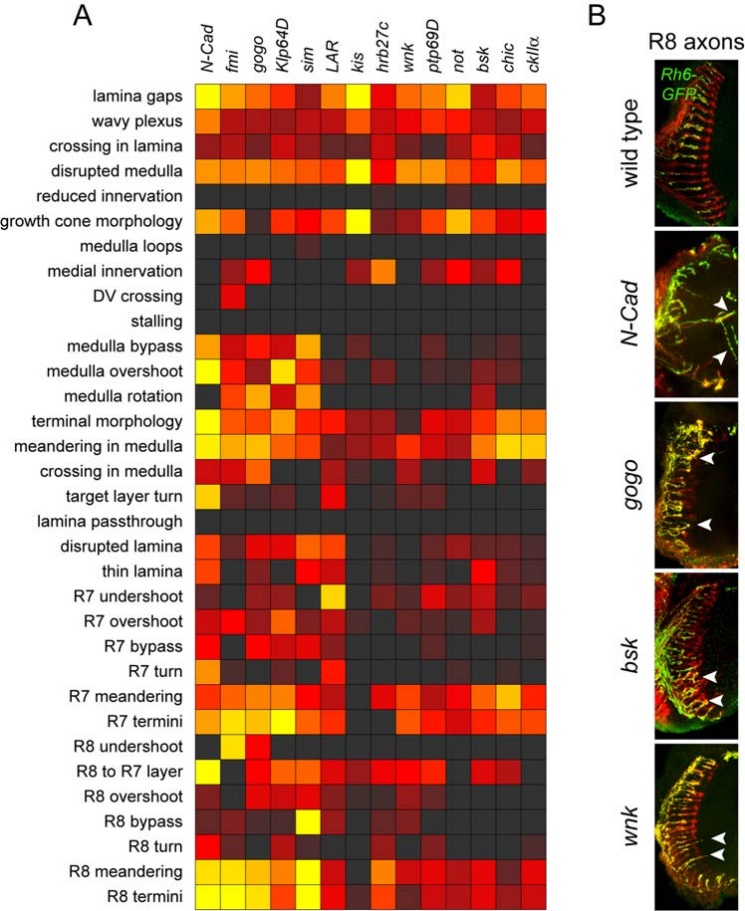
Medulla targeting genes. (A) Phenotypic analysis of mutants in the medulla targeting class, scored for all defect criteria as in Figure 1A. (B) Horizontal confocal sections of adult optic lobes, stained with anti-GFP to visualize R8 axons expressing an *Rh6-mCD8-GFP* reporter (green) and mAb24B10 to visualize all R axons (red). Animals carried whole-eye *eyFLP* clones of the indicated mutants. Arrowheads indicate R8-axons that overshoot their correct target layer and extend to or beyond the R7 target layer.

We isolated mutations in 14 genes for which the most pronounced defect is aberrant targeting of R7 and R8 axons in the medulla (Figures 1A and 5). Most of these mutations result in a general disorganization of medulla projections, including an irregular spacing of R7 and R8 axons. As for the lamina targeting cluster, the set of genes in this group encode a diverse set of molecules, including proteins involved in gene regulation, axonal transport, cell–cell interactions, and intracellular signalling. Cell signalling molecules are more prominent in the medulla targeting cluster than in the lamina targeting cluster. This may be due to mutations in these genes displaying less dramatic effects than those involved in protein synthesis or transport, and such subtle defects are more apparent in the fine arrangement of R7 and R8 projections in the medulla than in the crowded field of R1–R6 axons in the lamina.

Four genes in this cluster are involved in gene expression or protein transport: *kismet*, which encodes a chromatin remodelling factor [70], *single-minded*, encoding a bHLH-PAS domain transcription factor [71], *Hrb27c*, encoding an RNA-binding protein implicated in pre-mRNA splicing [72] and mRNA localization [73], and *Klp64D*, encoding a member of the kinesin-2 family of heterotrimeric kinesins [74],[75].

All five of the genes identified from our screen that encode cell surface proteins fall into the medulla targeting cluster. This includes two Cadherin genes, *N-cadherin* [76] and *flamingo* [77], and two receptor tyrosine phosphatase genes, *Ptp69D* and *LAR* [78]. Detailed phenotypic analyses of these genes have been presented previously, by us [4],[10],[12] or the Zipursky lab [3],[13],[15],[16]. The fifth gene, which we call *golden goal* (*gogo*), encodes a novel single-pass transmembrane protein with extracellular region that includes a single Thrombospondin Type I and a single CUB domain. Both of these domains are also found in other proteins involved in axon guidance, such as the Neuropilin [79] and Unc-5 family receptors [80]. The cytoplasmic region of the putative Gogo protein does not contain any known protein domain or catalytic activity. *gogo* mutant clones result in a severe disruption of R axon projections in the medulla (Figure 5B and [20]), which we could rescue with a *GMR-gogo* transgene (Table 2). It is interesting to note that the *gogo* phenotype clusters closely with *flamingo* (Figure 5), potentially suggesting a function in a common or related guidance mechanism [20].

The remaining five genes in this cluster encode putative cytoplasmic signalling molecules. These are *non-stop* (*not*), a protein deubiquitinating enzyme [49], *chickadee*, which encodes Profilin [81], and three members of the serine-threonine kinase superfamily: *basket* [82],[83], *casein kinase IIα* (*ckIIα*) [84],[85], and *wnk*. The role of *chic* in axon guidance has been well documented in numerous systems [9],[14],[17],[23],[86],[87]. As *not* is known to be required for the migration of the lamina glia, and thus indirectly for targeting of R1–R6 axons to the lamina, we wondered whether *not* mutant was picked up due to occasional clones in the lamina or a true R-cell autonomous role [49]. We did not observe defects in the migration of the lamina glia in *eyFLP* clones of our *not* alleles, and we could restore normal R-axon projections with a *GMR-not* transgene that expresses *not* exclusively in the eye disc (Table 2). We conclude that *not* has both autonomous and non-autonomous roles in R-axon targeting.

*bsk*, which encodes Jun N-terminal kinase, and *ckIIα*, which encodes the catalytic subunit of casein kinase II, have been shown to function in a variety of developmental processes. Functions of *bsk* include various aspects of cellular morphogenesis, such as dorsal closure and planar cell polarity [88]. A role for *bsk* in R axon pathfinding has been suggested from experiments using dominant negative constructs [41]. However, the specific topographic errors observed in these experiments do not match well with the general disorganization in the medulla that we observed in *bsk* mutant clones allele (Figure 5A). Functions of casein kinase are even more diverse, reflecting perhaps a wider range of substrates that includes the developmental proteins Cactus, Dishevelled, Antennapedia, and Enhancer of Split proteins [89]–[91]. Casein kinase II is a critical component of the circadian clock [92], and a function in axon pathfinding has not previously been reported. We confirmed an R-cell autonomous role for casein kinase in establishing axon projections in rescue experiments using a *GMR-ckIIα* transgene (Table 2).

The *wnk* gene encodes the single *Drosophila* member of a recently discovered and more enigmatic family of kinases, represented in mammals by the four kinases WNK1-4. This family of serine-threonine kinases is distantly related to the Ste20-like kinases, and owes its inappropriate name (With No Lysine [K]) to the fact that the lysine required for phosphoryl transfer lies in a different position to all other protein kinases (kinase subdomain I rather than subdomain II) [93]. The best characterised role of mammalian WNKs is in the regulation of electrolyte homeostasis, and mutations in two of the WNKs have been linked to hypertension [94]. Additionally, WNK1 functions in synaptogenesis by phosphorylation of Synaptotagmin2 [95]. Our *wnk* alleles carry mutations either within or C-terminal to the kinase domain, suggesting that Wnk's function in R-axon targeting requires its kinase domain in addition to its long and poorly conserved C-terminal region. We could rescue the *wnk* mutant phenotype with a genomic transgene, confirming the role of *wnk* in R-axon targeting (Table 2).

### General Remarks

We observed several mutants that have striking R-axon guidance phenotype in larvae but less severe phenotype in adults, indicating a transient nature of the defect. This is particularly evident in *tai, kis, not*, *enok, Br140, cdk8* and *wnk* phenotypes (Figures 1, 2, 3, 4, 5). One possible explanation for the discrepancy between adult and larval phenotypes is that different mechanisms underlie the development of the patterning of both systems. For example, a recent study of *gogo* function suggested that larval bundling defects are unrelated to the later defects seen in target recognition by R8-axons [20]. Another explanation could be that these mutants still retain the lamina cartridge formation defects even in the adult, but other more discerning assays would be needed. Analysis of R1–6 superposition defects in the lamina targeting neurons in adult in these mutants might be informative.

### Concluding Remarks

We began this study [4] at a time when relatively little was known about the molecular mechanisms of neuronal wiring in the *Drosophila* visual system [96] and before the completion of the *Drosophila* genome sequence [97]. Our long-term goal was to systematically identify as many as possible of the genes required for axon growth, guidance, and connectivity in this model system. Initial progress in gene identification was encouraging [4], [9]–[12], but slow, prompting us to develop methods for SNP mapping in *Drosophila* [7].

Using this method, we have been able to identify nearly all of the genes displaying guidance defects in our screen, including those represented by just a single allele. In most cases, the genetic lesion has been mapped to a single base pair. Our systematic identification of the genes and detailed characterisation of associated mutant phenotypes will serve as a springboard for further mechanistic studies of visual system wiring. Importantly, our work also demonstrates the feasibility of large-scale positional cloning in *Drosophila*. The large-scale mutagenesis screen has long been the trademark of *Drosophila* genetics, and indeed is one of its major strengths. Using approaches such as ours, systematic mutant recovery can now be augmented with systematic gene identification.

## Materials and Methods

### Genetics

Mutations were generated [4] and mapped [7] as described previously. In the first phase of recombination mapping, SNP genotypes were mostly determined by PLP assays [7], and in the second phase by DNA sequencing. Mapping in this second phase generally involved testing for the lethality of heteroallelic combinations, or, in the case of single alleles, failure to complement an existing deficiency. If a suitable deficiency was not available, fine mapping was performed using stocks containing two flanking EP elements [98] that had been placed in *cis*. Existing mutants, deficiency stocks, and EP elements were obtained from either the Bloomington or the Szeged stock centers. For sequencing, we extracted DNA from single embryos, identified homozygous mutant embryos by PCR with PLP primers [7], pooled their genomic DNA, PCR, sequenced the coding region and compared it to the parental reference chromosome.

### Rescue Constructs

The *wnk* genomic rescue transgene consisted of a 22 kb Asp718 fragment isolated from BACRP98-26P10 that was cloned into a pCaSpeR4 vector. *GMR* or *eyeless* rescue constructs were generated using standard PCR cloning techniques, using either genomic fragments containing small introns or full-length cDNAs as templates. For *hdc*, we used the long isoform amplified from *UAS-hdc* ^CAA^ [99]. For *dri*, *brat*, *ckIIα*, *not*, *cdk8*, and *unc-104*, genomic regions from the start to stop codons were amplified from genomic DNA. The *gogo* coding region was amplified from the EST clone RE53634, and *enok* from a full-length cDNA provided by Liqun Luo. *Br140* was cloned as an EcoRI fragment from the EST clone GH12223.

### Histology

Tangential eye sections, adult head sections, whole-mount adult brains, and whole-mount larval eye-brain complexes were prepared and stained as described previously [4],[10],[12]. Primary antibodies used were mAb24B10 (1∶50, [100]), rabbit anti-β-galactosidase (1∶2500, Cappel), and rabbit anti-GFP (1∶100–300, Torrey Pines). Secondary antibodies used were goat anti-rabbit Alexa-488 and goat anti-mouse Alexa-568 (1∶250 each, Molecular Probes). All fluorescent samples were mounted in Vectashield (Vector Laboratories). Head section stainings were performed manually for the initial characterisation, and using a Dako Autostainer plus (Dako Cytomation) for mapping and rescue experiments. Confocal images were acquired on Zeiss LSM 510 Axiovert 200M or LSM 510 Axioplan 2, or Leica SP2.

### Phenotypic Classification

Samples were scored for each phenotypic criteria on a 0 (wild-type) to 4 (most severe) scale according to the scale described in the Table S1. For examination of confocal images with LSM5 Image Examiner or Leica LCS lite, the final score was the average from 2–5 preparations. For larval omb-τlacZ, adult glass-lacZ sections and adult Rh1-τlacZ sections were examined under normal light microscope. Sections from 10–20 heads were examined for each allele. For omb-τlacZ stainings, we examined around 50 hemispheres for each allele scored. All mutants were scored independently by T.S. and J.B. and averaged. The genes for which multiple alleles were scored were averaged. Data were clustered using a *k*-means clustering algorithm [101], with manual adjustment and transformed into heat map using MS Excel macro function (Designed by Georg Dietzl). The range of phenotypic scores was calculated as the subtraction of the lowest score from the highest score among the samples from the same mutant allele for each criterion. These are shown for confocal samples to provide the tendency of expressivity of the phenotype. The range of scores for two individuals was averaged and transformed into color heat maps. For the scores quantified and averaged from more than 10 samples at the same time (omb-τlacZ samples, adult DAB sections and “lamina pass through”) a range was not given, however, the score itself gives the idea of penetration of the phenotype.

## Supporting Information

Figure S1 Expressivity and penetration of the phenotypic defects. (A) Color coded panels showing the range of values for each score for all the defect criteria and mutants shown in Figures 2–5. The range is shown on a scale from 0 (white: no variability) to 4 (blue: highly variable). The alleles and the genes are the same as shown in Figures 2–5. (B) Color coded panels showing the penetrance of the defects for each score for all the defect criteria and mutants shown in Figures 2–5. The penetrance is shown in 3 colors, red (fully penetrant), pink (partially penetrant) and white (no penetrance). If all the scores from all the samples from two scorers were never scored as wild type, it was defined as “fully penetrant”. Vice versa if everything is “0”, it is “no penetrance”. All other variations of scores were counted as “partially penetrant”. The alleles and the genes are the same as shown in A.
(20.32 MB TIF)

Table S1 Defect criteria.
(0.16 MB DOC)

Table S2 Scoring criteria for each defect criteria.
(0.11 MB DOC)
