# Supplementary figures and images for: Systematic Identification of Genes that Regulate Neuronal Wiring in the Drosophila Visual System
Source: PLoS Genet. 2008 May 30;4(5):e1000085. doi: 10.1371/journal.pgen.1000085 (PMC2377342; doi:10.1371/journal.pgen.1000085)

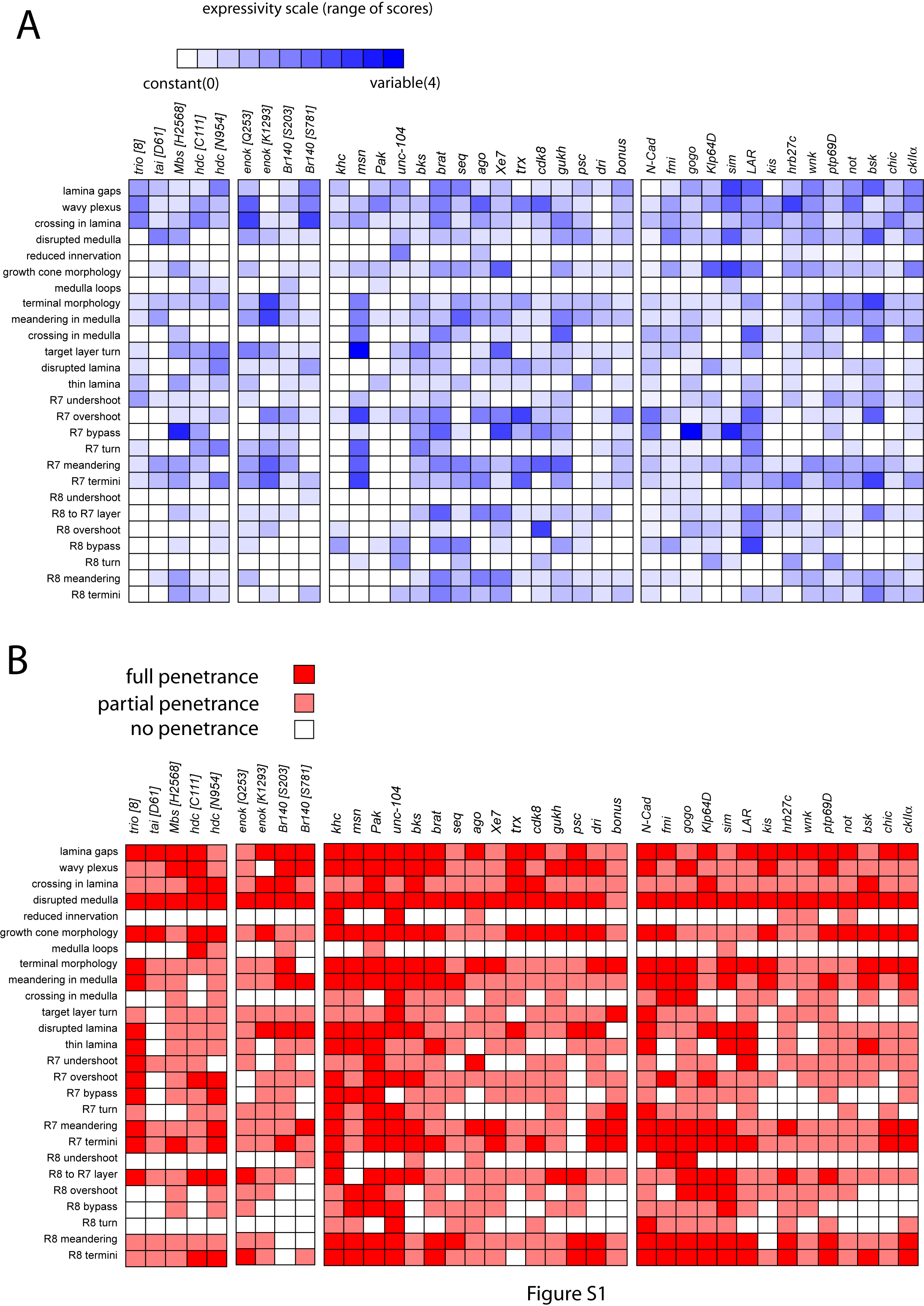

Supplement: Figure S1 — Expressivity and penetration of the phenotypic defects. (A) Color coded panels showing the range of values for each score for all the defect criteria and mutants shown in Figures 2– 5. The range is shown on a scale from 0 (white: no variability) to 4 (blue: highly variable). The alleles and the genes are the same as shown in Figures 2– 5. (B) Color coded panels showing the penetrance of the defects for each score for all the defect criteria and mutants shown in Figures 2– 5. The penetrance is shown in 3 colors, red (fully penetrant), pink (partially penetrant) and white (no penetrance). If all the scores from all the samples from two scorers were never scored as wild type, it was defined as “fully penetrant”. Vice versa if everything is “0”, it is “no penetrance”. All other variations of scores were counted as “partially penetrant”. The alleles and the genes are the same as shown in A. (20.32 MB TIF) [file pgen.1000085.s001.tif]
