# Supplementary material for: Systematic Identification of Genes that Regulate Neuronal Wiring in the Drosophila Visual System
Source: PLoS Genet. 2008 May 30;4(5):e1000085. doi: 10.1371/journal.pgen.1000085 (PMC2377342; doi:10.1371/journal.pgen.1000085)
Supplement: Table S1 — Defect criteria. (0.16 MB DOC) [file pgen.1000085.s002.doc]

| **Supplementary Table 1.** Defect criteria | | | | | | | | | |
| --- | --- | --- | --- | --- | --- | --- | --- | --- | --- |
|  |  |  |  |  | **Score** | | | | |
| **Defect criteria** | **Stage** | **Preparation** | **Marker** | **Description** | **0** | **1** | **2** | **3** | **4** |
| lamina gaps | larva | whole mount | mAb 24B10 | Discontinuous structure of lamina plexus | normal | weak | medium | strong | severe |
| wavy plexus | larva | whole mount | mAb 24B10 | Premature termination of axons in lamina, resulting in wavy plexus | normal | weak | medium | strong | severe |
| crossing in lamina | larva | whole mount | mAb 24B10 | Crossing of axons between the optic stalk and the lamina plexus | 0** | 1** | 2 – 5** | 5 – 10** | >10** |
| disrupted medulla | larva | whole mount | mAb 24B10 | Disrupted organisation of axons bundles and termini in the medulla | normal | weak | medium | strong | severe |
| reduced innervation | larva | whole mount | mAb 24B10 | Reduced number of axons innervate the lamina and medulla, even at late larval stages | normal | weak | medium | strong | severe |
| growth cone morphology | larva | whole mount | mAb 24B10 | Growth cones in the medulla have an abnormal spine or club shape, rather than the usual hand-like shape | normal | weak | medium | strong | severe |
| medulla loops | larva | whole mount | mAb 24B10 | Axons turn in the medulla and are re-routed back towards the lamina plexus | normal | weak | medium | strong | severe |
| medial innervation | larva | whole mount | omb-τlacZ | Some polar axons innervate a region close to the equator | 0 – 5% | 5 – 10% | 10 – 25% | 25 – 50% | >50% |
| DV crossing | larva | whole mount | omb-τlacZ | Some polar axons do cross to the other side of the optic lobe, but generally remain polar | 0 – 5% | 5 – 10% | 10 – 25% | 25 – 50% | >50% |
| stalling | larva | whole mount | omb-τlacZ | Axons stall in the optic stalk or at the surface of the optic lobe | 0 – 5% | 5 – 10% | 10 – 25% | 25 – 50% | >50% |
| medulla bypass | adult | sections | glass-lacZ | Anterior or posterior axons extend around edges of medulla | normal | weak | medium | strong | severe |
| medulla overshoot | adult | sections | glass-lacZ | Axons extend through the medial regions of the medulla | 0** | 1** | 2 – 5** | 5 – 10** | >10** |
| medulla rotation | adult | sections | glass-lacZ | Medulla incompletely rotated | 0 – 10° | 10 – 15° | 15 – 30° | 30 – 45° | >45° |
| terminal morphology | adult | whole mount | mAb 24B10 | Axon termini have an abnormal morphology | normal | weak | medium | strong | severe |
| meandering in medulla | adult | whole mount | mAb 24B10 | Irregular spacing and fasciculation of axons in the medulla | normal | weak | medium | strong | severe |
| crossing in medulla | adult | whole mount | mAb 24B10 | Crossing of axon bundles in the medulla | 0* | 1* | 2 – 5* | 5 – 10* | >10* |
| target layer turn | adult | whole mount | mAb 24B10 | Axons do not stop at their target layer, but turn and extend perpendicular to it | 0* | 1* | 2 – 5* | 5 – 10* | >10* |
| lamina passthrough | adult | sections | Rh1-τlacZ | R1 – R6 axons continue through the lamina and into the medulla | 0 – 0.5** | 0.5 – 2** | 2 – 5** | 5 – 10** | >10** |
| disrupted lamina | adult | sections | Rh1-τlacZ | Disrupted organisation of lamina columns | normal | weak | medium | strong | severe |
| thin lamina | adult | sections | Rh1-τlacZ | Reduced lamina width | 20 – 25mm | 15 – 20mm | 10 – 15mm | 5 – 10mm | 0 – 5mm |
| R7 undershoot | adult | whole mount | Rh4-GFP | R7 axons stop before reaching their medulla target layer | 0* | 1* | 2 – 5* | 5 – 10* | >10* |
| R7 overshoot | adult | whole mount | Rh4-GFP | R7 axons extend beyond their normal medulla target layer | 0* | 1* | 2 – 5* | 5 – 10* | >10* |
| R7 bypass | adult | whole mount | Rh4-GFP | R7 axons extend around edges of medulla | normal | weak | medium | strong | severe |
| R7 turn | adult | whole mount | Rh4-GFP | R7 axons do not stop at their target layer, but turn and extend perpendicular to it | 0* | 1* | 2 – 5* | 5 – 10* | >10* |
| R7 meandering | adult | whole mount | Rh4-GFP | Irregular spacing and fasciculation of R7 axons in the medulla | normal | weak | medium | strong | severe |
| R7 termini | adult | whole mount | Rh4-GFP | R7 termini in the medulla have an abnormal spine or club shape | normal | weak | medium | strong | severe |
| R8 undershoot | adult | whole mount | Rh6-GFP | R8 axons stop before reaching their medulla target layer | 0* | 1* | 2 – 5* | 5 – 10* | >10* |
| R8 to R7 layer | adult | whole mount | Rh6-GFP | R8 axons terminate in the R7 target layer in the medulla | 0* | 1* | 2 – 5* | 5 – 10* | >10* |
| R8 overshoot | adult | whole mount | Rh6-GFP | R8 axons extend beyond the normal R7 target layer in the medulla | 0* | 1* | 2 – 5* | 5 – 10* | >10* |
| R8 bypass | adult | whole mount | Rh6-GFP | R8 axons extend around edges of medulla | normal | weak | medium | strong | severe |
| R8 turn | adult | whole mount | Rh6-GFP | R8 axons do not stop at their target layer, but turn and extend perpendicular to it | 0* | 1* | 2 – 5* | 5 – 10* | >10* |
| R8 meandering | adult | whole mount | Rh6-GFP | Irregular spacing and fasciculation of R8 axons in the medulla | normal | weak | medium | strong | severe |
| R8 termini | adult | whole mount | Rh6-GFP | R8 termini in the medulla have an abnormal spine or club shape | normal | weak | medium | strong | severe |

* per 30μm confocal stack: Around 130 axon bundles. With R7 or R8 specific markers, around 90 axons.

** per hemisphere
