# Supplementary material for: Systematic Identification of Genes that Regulate Neuronal Wiring in the Drosophila Visual System
Source: PLoS Genet. 2008 May 30;4(5):e1000085. doi: 10.1371/journal.pgen.1000085 (PMC2377342; doi:10.1371/journal.pgen.1000085)
Supplement: Table S2 — Scoring criteria for each defect criteria. (0.11 MB DOC) [file pgen.1000085.s003.doc]

| **Supplementary Table 2.** Scoring criteria for each defect criteria | | | |
| --- | --- | --- | --- |
|  | **Score** | | **Further explanation** |
| **Defect criteria** | **0 (lowest)** | **4 (highest)** |
| lamina gaps | normal | severe | The length of the disrupted lamina was assessed and expressed as percentage of the total lamina length. Normal (no lamina disruption), weak (5-10% of the lamina was disrupted), medium (10-25%), strong (25-40%), severe (>40%). |
| wavy plexus | normal | severe | The area occupied by prematurely terminating axons was assessed and expressed as percentage of the total lamina axon termination zone. Normal (no premature termination), weak (5-10% of the lamina termination zone affected), medium (10-25%), strong (25-40%), severe (>40%). |
| crossing in lamina | 0** | >10** | The number of crossing axons was determined. Normally 35-50 axon fascicles could be distinguished per examined hemisphere. |
| disrupted medulla | normal | severe | Axon termini in medulla form evenly spaced array pattern. The strength of the phenotype was subdivided into 5 categories by the visual impression. |
| reduced innervation | normal | severe | The number of axons innervating the lamina and medulla was visually assessed and expressed as percentage of an average wild type sample (around 40 axon bundles). Weak (around 80% axons innervateing), medium (about 70%), strong (about 60%), severe (less than 50%). |
| growth cone morphology | normal | severe | The number of the growth cones in the medulla with a disrupted morphology was assessed and expressed as percentage of matured medulla growth cones (around 60 growth cones). Normal (no growth cone disruption), weak (5-10% of the labelled growth cones was disrupted), medium (10-25%), strong (25-40%), severe (>40%). |
| medulla loops | normal | severe | The number of axons that aberrantly turn in the medulla and head backwards to the lamina plexus was assessed and expressed as percentage of all labelled growth cones. Normal (no loops), weak (occasionally seen), medium (1-2 axons), strong (2-5 axons), severe (more than 5 per sample) |
| medial innervation | 0 – 5% | >50% | The number of polar axons aberrantly innervating the equator zone was assessed and expressed as percentage of all brain hemisphere. "xy%" means xy% of hemisphere has axon bundle(s) innervating the medial zone. More than 50 samples were quantified for each allele. |
| DV crossing | 0 – 5% | >50% | The number of polar axons aberrantly crossing the equator and/but innervating polar regions on the other side of the optic lobe was assessed and expressed as percentage of all brain hemispheres. More than 50 samples were quantified for each allele. |
| stalling | 0 – 5% | >50% | The number of polar axons aberrantly stalling in the optic stalk or at the surface of the optic lobe was assessed and expressed as percentage of all brain hemispheres counted. The number of polar axons aberrantly innervating the equator zone was assessed and expressed as percentage of all brain hemisphere. More than 50 samples were quantified for each allele. |
| medulla bypass | normal | severe | The strength of the phenotype was subdivided into 5 categories by the visual impression. Normal (no bypass), weak (occasionally thin axon bundle bypass is seen), medium (often the bypass is seen), strong (the bypassing axon form medium bundle and in most of the sample), severe (The bypassing axons form thick bundle in every sample) |
| medulla overshoot | 0** | >10** | The number of axon bundles extending through the medial medulla region was determined. |
| medulla rotation | 0 – 10° | >45° | The angular deviation from a complete rotation was assessed and expressed in averaged angular degrees from around 10 samples for each allele. |
| terminal morphology | normal | severe | The number of the growth cones with a disrupted morphology was assessed and expressed as percentage of all labelled growth cones. Normal (no growth cone disruption), weak (5-10% of the labelled growth cones was disrupted), medium (10-25%), strong (25-40%), severe (>40%). |
| meandering in medulla | normal | severe | The area covered by axons with irregular spacing and fasciculation was assessed and expressed as percentage of the entire area covered by axons innervating the medulla. Normal (<2%), weak (>2-15% axons innervateing), medium (>15-30%), strong (>30-45%), severe (>=45%). |
| crossing in medulla | 0* | >10* | The number of crossing axon bundles was determined. |
| target layer turn | 0* | >10* | The number of non-stopping and misrouting axon bundles was determined in given sample. |
| lamina passthrough | 0 – 0.5** | >10** | The number of overshooting R1-R6 axon bundles was determined. Normally 200 axon fascicles could be distinguished per examined hemisphere. |
| disrupted lamina | normal | severe | The area of lamina columns with a disrupted organization was assessed and expressed as percentage of the entire area of lamina columns. Normal (<5% of the lamina columns area disrupted), weak (>5-15%), medium (>15-30%), strong (>30-45%), severe (>=45%). |
| thin lamina | 20 – 25mm | 0 – 5mm | The lamina width was assessed and the samples and the average was scored for each allele. Normal (lamina width of 20-25mm), weak (15-20mm), medium (10-15mm), strong (5-10mm), severe (0-5mm). |
| R7 undershoot | 0* | >10* | The number of R7 axons stopping before reaching their target layer in the medulla was determined. |
| R7 overshoot | 0* | >10* | The number of R7 axons extending beyond their target layer in the medulla was determined. |
| R7 bypass | normal | severe | The strength of the phenotype was subdivided into 5 categories by the visual impression. Normal (no bypass), weak (occasionally thin axon bundle bypass is seen), medium (often the bypass is seen), strong (the bypassing axon form medium bundle and in most of the sample), severe (The bypassing axons form thick bundle in every sample) |
| R7 turn | 0* | >10* | The number of overshooting and misrouting R7 axon bundles was determined. |
| R7 meandering | normal | severe | The area covered by R7 axons with irregular spacing and fasciculation was assessed and expressed as percentage of the entire area covered by innervating R7 axons. Normal (<5%), weak (>5-15% axons innervateing), medium (>15-30%), strong (>30-45%), severe (>=45%). |
| R7 termini | normal | severe | The number of R7 growth cones with a disrupted morphology was assessed and expressed as percentage of all labeled R7 growth cones. Normal (no growth cone disruption), weak (5-10% of the labeled growth cones was disrupted), medium (10-25%), strong (25-40%), severe (>40%). |
| R8 undershoot | 0* | >10* | The number of prematurely stopping R8 axon bundles was determined. |
| R8 to R7 layer | 0* | >10* | The number of R8 axon bundles that target the R7 layer was determined. |
| R8 overshoot | 0* | >10* | The number of R8 axon bundles that overshoot the R7 layer was determined. |
| R8 bypass | normal | severe | The strength of the phenotype was subdivided into 5 categories by the visual impression. Normal (no bypass), weak (occasionally thin axon bundle bypass is seen), medium (often the bypass is seen), strong (the bypassing axon form medium bundle and in most of the sample), severe (The bypassing axons form thick bundle in every sample) |
| R8 turn | 0* | >10* | The number of overshooting and misrouting R8 axon bundles was determined. |
| R8 meandering | normal | severe | The area covered by R8 axons with irregular spacing and fasciculation was assessed and expressed as percentage of the entire area covered by innervating R8 axons. Normal (<2%), weak (>2-15% axons innervating), medium (>15-30%), strong (>30-45%), severe (>=45%). |
| R8 termini | normal | severe | The number of R8 growth cones with a disrupted morphology was assessed and expressed as percentage of all labeled R8 growth cones. Normal (no growth cone disruption), weak (5-10% of the labeled growth cones was disrupted), medium (10-25%), strong (25-40%), severe (>40%). |

* per 30mm confocal stack: Around 130 axon bundles. With R7 or R8 specific markers, ~90 axons.

** per hemisphere
